# Three-year evaluation of a program teaching social determinants of health in community-based medical education: a general inductive approach for qualitative data analysis

**DOI:** 10.1186/s12909-023-04320-2

**Published:** 2023-05-12

**Authors:** Sachiko Ozone, Junji Haruta, Ayumi Takayashiki, Takami Maeno, Tetsuhiro Maeno

**Affiliations:** 1 Department of Family Medicine, Institute of Medicine, General Practice and Community Health, University of Tsukuba, 1-1-1 Tennodai, Tsukuba, Ibaraki 305-8575 Japan; 2 Department of Primary Care and Medical Education, Institute of Medicine, University of Tsukuba, 1-1-1 Tennodai, Tsukuba, Ibaraki 305-8575 Japan; 3 Medical Education Center, School of Medicine, Keio University, 35 Shinanomachi, Shinjuku, Tokyo, 160-8582 Japan

**Keywords:** Social determinants of health, Community-based medical education, Reflection, Undergraduate medical education

## Abstract

**Background:**

Social determinants of health (SDH) are intricately intertwined with various social and economic factors. Reflection is essential for learning about SDH. However, only a few reports have focused on reflection in SDH programs; most were cross-sectional studies. We aimed to longitudinally evaluate a SDH program in a community-based medical education (CBME) curriculum that we introduced in 2018 based on the level of reflection and content on SDH in students’ reports.

**Methods:**

Study design: General inductive approach for qualitative data analysis. Education program: A 4-week mandatory clinical clerkship in general medicine and primary care at the University of Tsukuba School of Medicine in Japan was provided to all fifth- and sixth-year medical students. Students underwent a 3-week rotation in community clinics and hospitals in suburban and rural areas of Ibaraki Prefecture. After a lecture on SDH on the first day, students were instructed to prepare a structural case description based on encounters during the curriculum. On the final day, students shared their experiences in a small group session and submitted a report on SDH. The program was continuously improved and faculty development was provided. Study participants: Students who completed the program during October 2018–June 2021. Analysis: Levels of reflection were categorized as reflective, analytical, or descriptive. The content was analyzed based on the Solid Facts framework.

**Results:**

We analyzed 118 reports from 2018–19, 101 reports from 2019–20, and 142 reports from 2020–21. There were 2 (1.7%), 6 (5.9%), and 7 (4.8%) reflective reports; 9 (7.6%), 24 (23.8%), and 52 (35.9%) analytical reports; and 36 (30.5%), 48 (47.5%), and 79 (54.5%) descriptive reports, respectively. The others were not evaluable. The number of Solid Facts framework items in reports were 2.0 ± 1.2, 2.6 ± 1.3, and 3.3 ± 1.4, respectively.

**Conclusions:**

Students’ understanding of SDH deepened as the SDH program in the CBME curriculum improved. Faculty development might have contributed to the results. Reflective understanding of SDH might require more faculty development and integrated education of social science and medicine.

**Supplementary Information:**

The online version contains supplementary material available at 10.1186/s12909-023-04320-2.

## Background

Social determinants of health (SDH) are non-medical factors that influence health outcomes, including environments where people are born, grow, work, live, and age [1]. SDH have a substantial impact on people’s health, and healthcare interventions alone cannot affect the health impact of SDH [1–3]. Healthcare professionals are expected to learn about SDH [4, 5] and contribute to society as health advocates [6] to mitigate the negative effects of SDH [4–6].

The importance of teaching about SDH in undergraduate medical education has been widely recognized [4, 5, 7], but there are many issues related to SDH education. It is essential for medical students to relate SDH to biological pathways of disease [8], which might be more familiar, but the connection between SDH education and clinical training might remain limited. SDH education was provided more in the first and second years of undergraduate medical education than in the third or fourth years as reported by the American Medical Association’s Accelerating Change in Medical Education Consortium [7]. Not all medical schools in the United States teach about SDH in the clinical phase [9], program durations vary [10], and programs are often elective [5, 10]. With a lack of agreement on competency in SDH, learner assessment and program evaluation strategies are also diverse [9]. In order to promote SDH education in undergraduate medical education, implementation of a SDH program in the latter years of undergraduate medical education and appropriate program evaluations are needed [7, 8]. In Japan, the importance of SDH education in medical education has also been recognized. SDH education was introduced into the model core curriculum for medical education, which indicates the goals to be achieved at graduation from medical school, in 2017 [11]. It has been further emphasized in the 2022 revision [12]. However, SDH education methods and evaluations have not been established in Japan.

In our previous studies, we reported on SDH program evaluation in the community-based medical education (CBME) curriculum in upper-year medical students from a university in Japan [13] by evaluating levels of reflection in their reports and the process by which they learn about SDH [14]. Understanding SDH requires transformative learning [10]. Studies, including ours, focused on learners’ reflections to evaluate SDH programs [10, 13]. In the initial program we provided, students seemed to learn some factors of SDH more than others and their levels of reflection on SDH were relatively low [13]. Students deepened their understanding of SDH through experiences in the community that transformed their perspective of the medical model into a life model [14]. These findings were valuable when curricular standards for SDH education and its assessment and evaluation have not been sufficiently established [7]. However, longitudinal evaluations of undergraduate SDH programs have been rarely reported. If we could show the process of improving and evaluating SDH programs longitudinally, it would be an example for the development and evaluation of better SDH programs, which will contribute to developing standards and competencies of undergraduate SDH education.

The purpose of this study was to show the continuous program improvement process in an SDH education program for medical students and to longitudinally evaluate an SDH education program in a CBME curriculum by evaluating levels of reflection in students’ reports.

## Methods

### Study design

The study used a general inductive approach for qualitative data analysis of the program annually for 3 years. It evaluated SDH reports of medical students who participated in an SDH program in a CBME curriculum. The general inductive approach is a systematic procedure for analyzing qualitative data, one in which the analysis is likely to be guided by specific evaluation objectives. Its purpose is to enable the emergence of research outcomes from frequent, dominant, or significant themes inherent in the raw data, without being predetermined by structured methodologies [15].

### Population and settings

The participants of the study were fifth-year and sixth-year medical students in the University of Tsukuba School of Medicine who participated in a mandatory 4-week clinical clerkship in the CBME curriculum either between September 2018 and May 2019 (2018–19), September 2019 and March 2020 (2019–20), or October 2020 and July 2021 (2020–21).

### CBME curriculum

The structure of the 4-week CBME curriculum is comparable to those in our previous studies [13, 14]. The students participated in the CBME curriculum either in their fifth or sixth year as a part of an introduction to medicine course, which aimed to teach the essentials for healthcare professionals, including health promotion, professionalism, and interprofessional collaboration. The goals of the CBME curriculum were to familiarize students with the expertise of family physicians who provide appropriate care in various clinical settings; convey the health issues of citizens, patients, and families within the local healthcare system; and develop clinical reasoning skills. Every 4 weeks, 15–17 students participated in the course. The rotation included 1 week in community-based settings, 1–2 weeks in community clinics or small hospitals, up to 1 week in community hospitals, and 1 week in the family medicine department of the university hospital. On the first day and the last day, the students gathered at the university and attended lectures and small group discussions. The faculty explained the goals of the curriculum to the students on the first day. The students were required to submit a final report related to the goals of the curriculum. Three core faculty members (AT, SO, and JH) planned most of the CBME curriculum and the SDH program. The program was provided by both core faculty and 10–12 other faculty members who are either involved in undergraduate education at the university while providing CBME programs as practicing family physicians in the community or are non-physician medical faculty members familiar with CBME.

### SDH program

**Fig. 1:**
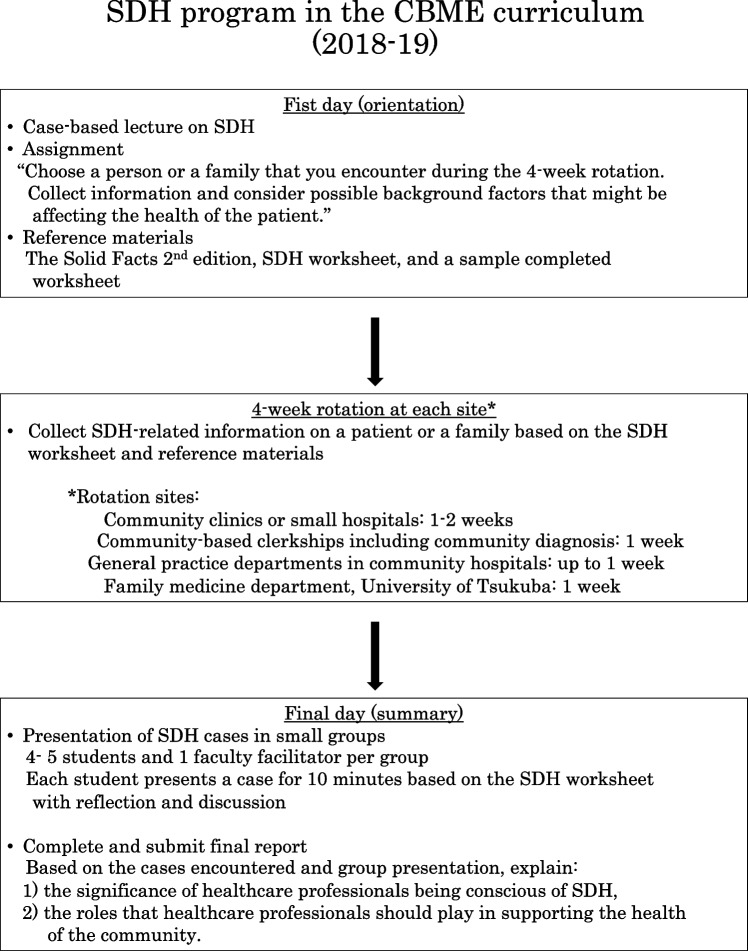

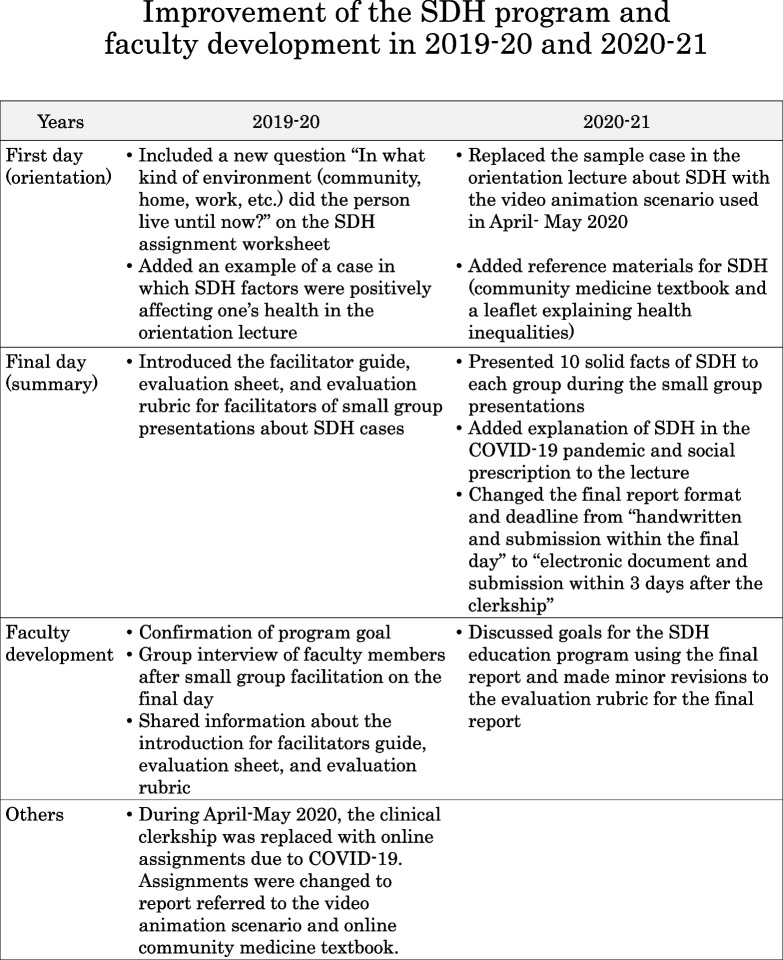
Overview of the SDH program in the CBME curriculum at the University of Tsukuba, School of Medicine in 2018–19 and the process for improving the SDH program and faculty development in 2019–20 and 2020–21. 2018–19 refers to the program from October 2018 to May 2019, 2019–20 refers to the program from October 2019 to March 2020, and 2020–21 refers to the program from October 2020 to June 2021. SDH: social determinant of health, COVID-19: coronavirus disease 2019

The structure of the SDH program in the CBME curriculum followed the structure of those in our previous studies [13, 14], with continuous modification (Fig. 1). On the first day, students attended a case-based lecture on SDH and were given a SDH assignment to complete during their 4-week rotation. The students were asked to choose a person or a family that they encountered during their clerkship and collect information to consider possible factors that may be affecting their health. The Solid Facts 2nd edition [15] from the World Health Organization, SDH worksheet, and samples of the completed worksheet were provided as reference materials. On the final day, students presented their SDH cases in small groups that contained 4–5 students and 1 faculty facilitator per group. After the presentations, the students were assigned to submit a final report on the CBME curriculum. They were instructed to describe their experiences in their 4-week rotation and relate it to their experiences; they were asked to explain 1) the significance of healthcare professionals being aware of SDH and 2) the roles that they should play in supporting the health of the community. Instructions for the report and a rubric for how the report will be evaluated were presented to the students (Supplementary materials). For student assessment, approximately 15 faculty members, which included the core faculty, evaluated reports based on the evaluation rubric.

### Improvement of the SDH program and faculty development (Fig. 1)

We continuously modified the SDH program and provided faculty development since it began in 2018. At the beginning of the program in 2018, the core faculty members who designed the program provided a faculty development lecture to other faculty members who would take part in the SDH program. The first faculty development lecture was on SDH and having sociological perspectives in clinical settings.

After the 2018–19 program, we held faculty development meetings to discuss and confirm the program’s goals and modified the program accordingly. For the 2019–20 program, which took place from September 2019 to March 2020, we introduced a facilitator guide, evaluation sheet, and evaluation rubric for faculty facilitators for the small group presentation of SDH cases on the final day. After each set of small group presentations, we conducted a group interview with faculty facilitators to reflect on the program.

In the third-year program from September 2020 to June 2021, we conducted faculty development sessions to discuss goals for the SDH education program using the final report. We made minor revisions to the final report assignment and its evaluation rubric (Supplementary materials). We also changed the format and deadline from handwritten and submission by the final day to electronic document and submission within 3 days after the clerkship.

### Analysis

As a method for deriving important and frequent themes inherent in the reports, we evaluated the levels of reflection in the description of SDH and extracted the Solid Facts factors that were mentioned. Since reflection has been considered as a form of education and program assessment in a previous review [10], we determined that evaluations of the level of reflection in reports could be used to evaluate the SDH program. Given that reflection is defined differently depending on the context, we adopted the definition of reflection in the context of medical education as “a process of analyzing, questioning, and reframing an experience in order to make an assessment of it for the purpose of learning and/or to improve practice” as described by Aronson based on Mezirow’s definition of critical reflection [16]. The levels of reflection in the 4-week final reports from 2018–19, 2019–20, and 2020–21 were categorized, like in our previous study [13], as descriptive, analytical, or reflective. This categorization was based on the style of academic writing as described by the University of Reading [17]. Since some educational studies have evaluated reflection levels in a similar manner [18], we determined that it was appropriate to use this categorization to evaluate the level of reflection in reports for this study. Descriptive reports were those that explained the case using the SDH framework but lacked integration of the factors. Analytical reports were those that integrated SDH factors. Reflective reports were those in which the author reflected further on his or her own ideas about SDH. Reports that did not fall into any of these categories were categorized as unable to be evaluated. We evaluated the SDH factors described in the reports using content analysis based on the Solid Facts framework, second edition [19]. The content of the final report corresponded to the goals of the program. Students were asked to reflect on their experiences in order to explain the significance of medical professionals being aware of SDH and their own roles in society. After SO analyzed the levels of reflection and the SDH factors described in the reports, SO, JH, and AT discussed and confirmed the criteria for the categories. SO repeated the analysis. SO, JH, and AT further discussed the analysis of the reports that required changes in categorization. They reached final consensus for the analysis of all reports.

### Ethics approval

This study was approved by the University of Tsukuba medical ethics board (No. 1434–2).

## Results

In 2018–19, 2019–20, and 2020–21, a total of 118, 101, and 142 students participated the SDH program, respectively. There were 35 (29.7%), 34 (33.7%), and 55 (37.9%) female students, respectively.

**Fig. 2:**
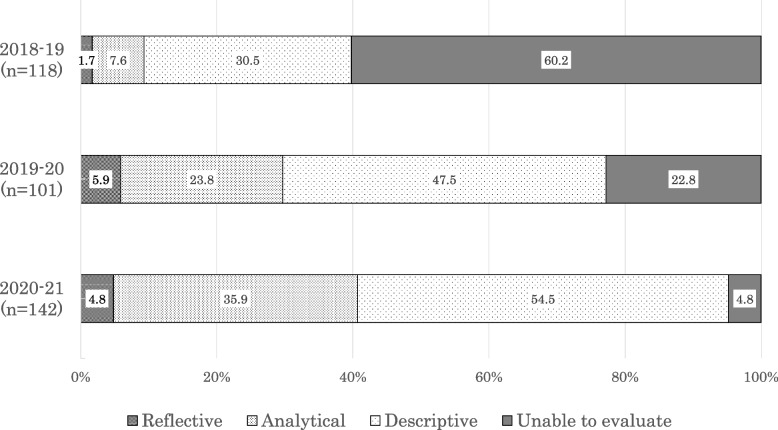
Levels of reflection in student reports from the SDH program provided in 2018–19, 2019–20, and 2020–21. 2018–19 refers to the program from October 2018 to May 2019, 2019–20 refers to the program from October 2019 to March 2020, and 2020–21 refers to the program from October 2020 to June 2021. SDH: social determinant of health

Figure 2 shows the distribution of levels of reflection for each year compared to our previous study, which analyzed the level of reflection in reports written by students in 2018–19 [13]. There were 36 (30.5%) reports categorized as descriptive in 2018–19, 48 (47.5%) in 2019–20, and 79 (54.5%) in 2020–21. There were 9 (7.6%) analytical reports in 2018–19, 24 (23.8%) in 2019–20, and 52 (35.9%) in 2020–21. There were 2 (1.7%) reflective reports in 2018–19, 6 (5.9%) in 2019–20, and 7 (4.8%) in 2020–21. There were 71 (60.2%) reports categorized as unable to be evaluated in 2018–19, 23 (22.8%) in 2019–20, and 7 (4.8%) in 2020–21. Table 1 shows examples of the reports for each level of reflection.

**Table 1:** Examples of reports that were evaluated as reflective, analytical, or descriptive

Level of reflection	Report content
Reflective	In each case, the social factors that affected health differed according to the patient, family environment, or region. Each factor affected each other and regulated the patient’s health conditions. I felt that if healthcare professionals were not conscious of this concept, we would not reach a fundamental solution to each patient’s problem. Considering that a patient’s life is shaped by various backgrounds such as family environment, human relationships, and their community, it can be said that the social determinants of health are rooted in the problems of the community itself, such as family and community conditions. Therefore, in order to support the health of the community as healthcare professionals, we must not only look at each patient, but also understand the characteristics, history, and culture of the community from a broader perspective and intervene in the problems of the community itself. Especially for people who belong to the community, its culture is taken for granted, so people from outside the community, governments, residents, etc. must gather and discuss from a wide range of perspectives to tackle the problem together
Analytical	For patients who have been living in such an environment for many years, there must be some social determinants of health that they feel are “normal” and not considered as problems, such as food or lifestyle. There must be cases where they become aware of it as a problem or get strong motivation for improvement only when healthcare professionals point it out. Healthcare professionals asking about the patient’s life in detail would lead to better treatment and a higher treatment success rate
Descriptive	In this clinical clerkship, I actually went to the community and met various patients. I thought that we could not support patients just by seeing those who come to the hospital. In the community, there are people who cannot come or have difficulty coming to the hospital. Not all patients in the community come to the hospital. Like the patients I met in this clerkship, I felt that local healthcare professionals need to be aware of not only their illness but also social factors such as their family and housing conditions

Legend: The reflective reports explained and compared multiple patients and discussed the relationship between SDH factors and explained ideas on the role of healthcare professionals in decreasing health inequity. The analytical reports explained the term SDH, discussed multiple SDH factors (mostly in a single patient), and focused on upstream factors. The descriptive reports explained the current condition of the patient using the SDH framework but lacked integration of the factors

*SDH* Social determinant of health

**Fig. 3:**
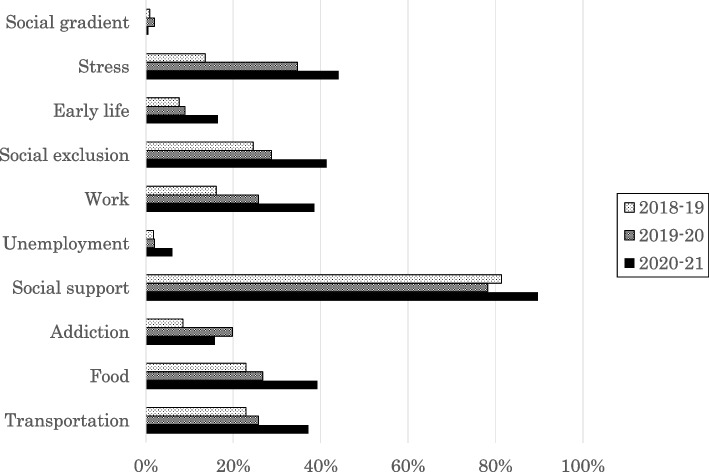
Percentage of students who mentioned each factor in the Solid Facts framework (second edition) in reports from 2018–19, 2019–20, and 2020–21. The period 2018–19 refers to October 2018 to May 2019, 2019–20 refers to October 2019 to March 2020, and 2020–21 refers to October 2020 to June 2021, which are the dates of the program. There were 118 students in 2018–19, 101 in 2019–20, and 142 in 2020–21

The percentage of SDH factors described in the reports is shown in Fig. 3. The mean number of factors described in reports was 2.0 ± 1.2 in 2018–19, 2.6 ± 1.3 in 2019–20, and 3.3 ± 1.4 in 2020–21.

## Discussion

We introduced the SDH education program into the compulsory CBME curriculum for upper-year medical students and presented the results of a 3-year program evaluation by assessing the levels of reflection about SDH in the students’ reports. After 3 years of program introduction and continuous program improvement, most students were able to describe SDH and explain some SDH factors in their reports. On the other hand, only a few students were able to write reflective reports about SDH.

Compared to 2018–19, the proportion of analytical and descriptive reports gradually increased and non-evaluable reports markedly decreased in 2019–20 and 2020–21, which might have resulted from improvements in the program and faculty development. Faculty development is essential for the SDH education program [4, 9]. We provided continuous faculty development to faculty members involved in the program. When the program started in 2018, the Japan Primary Care Association, one of the academic societies for family medicine and community health in Japan, had just issued a statement on SDH to primary care physicians in Japan. Most faculty members were unfamiliar with the term SDH. Faculty members gradually deepened their understanding of SDH by participating in the program and interacting with students at case presentations. In addition, clarification of goals for the SDH program through continuous faculty development might have contributed to the proficiency of the faculty members. One possible hypothesis might be that the program has improved over time. Sufficient time and effort might be needed for such program improvement. Regarding the 2020–21 program, the impact of the COVID-19 pandemic on the students’ own lives and education [20–23] might have led students to perceive SDH as issues affecting their own lives and helped them reflect on SDH.

Although the number of SDH factors mentioned in reports increased, the frequency of their appearance varied based on the factors, which may have been related to the characteristics of the practice setting. The high percentage for social support was unsurprising given frequent contact with patients already connected to medical care. Transportation was also frequently mentioned, which may be due to the fact that the CBME sites were located in suburban or rural areas, where students actually experienced inconvenient transportation conditions and had opportunities to interact with people in such environments. Stress, social exclusion, labor, and food were also mentioned, which more students could have encountered during their practice. On the other hand, the impact of social disparities and unemployment on health might have been difficult to understand during this short training period. The SDH factors that students can experience during their practice might also be influenced by the characteristics of the practicing field.

Our study was valuable in that we continuously evaluated the SDH program within the CBME program, which we provided to upper-year medical students, through assessment of the level of reflection in student reports. Upper-year medical students, who have been studying clinical medicine for several years, have a medicalized perspective. Therefore, they have the potential to learn by relativizing the social scientific perspective required by the SDH program and their own medical perspective [14]. Thus, providing an SDH program to these students is of great significance. In this study, we were able to conduct continuous program evaluation by evaluating the level of reflection in students’ reports. Campbell et al. reported that in U.S. medical schools and physician assistant programs, SDH program evaluation is conducted through surveys, focus groups, or mid- to group-level assessment data. The most common measures used in program evaluation were learner reactions and levels of satisfaction, learner knowledge, and learner behavior [9], but standardized and effective evaluation methods for SDH education programs have not yet been established. This study, which highlighted longitudinal changes in program evaluation along with continuous program improvement, will contribute to promoting SDH program development and evaluation at other educational institutions.

Although overall levels of reflection among students improved markedly throughout the study period, the proportion of students who wrote reflective reports remained low. Cultivation of more sociological perspectives might be needed for further improvement. The assignments in the SDH program required students to integrate sociological and medical perspectives, which had a different type of complexity compared to that of the medical model [14]. As we mentioned above, providing SDH programs to upper-year students is significant, but to organize and improve the education program starting from earlier years of medical education to nurture a sociological perspective along with the medical perspective and integration of both might be effective for further understanding of SDH. Further improving the faculty’s sociological perspective might also contribute to improving the students’ levels of reflection.

The study had several limitations. First, the study setting was limited to a single medical school in Japan, with the CBME setting limited to one region of Japan in sub-urban or rural areas, as in our previous studies [13, 14]. We have thoroughly explained the setting of this study and previous studies. Even with such limitations, it is noteworthy that we demonstrated the results of the SDH program in the CBME program over multiple years. Second, it was difficult to determine the possibility of implementing reflective learning beyond the SDH program from this study alone. Further investigation is warranted to promote reflective learning on SDH in undergraduate medical education. Third, whether faculty development contributed to program improvement is beyond the scope of the hypothesis in this study. The effectiveness of faculty development needs to be verified through further study.

## Conclusions

We conducted a longitudinal evaluation of an SDH education program for upper-grade medical students in a CBME curriculum. We showed that the students’ understanding of SDH deepened as the program improved. SDH program improvement might take time and effort, but faculty development to deepen the faculty’s understanding of SDH might be effective. In order to further enhance students’ understanding of SDH, it might be necessary to design the program in a way that integrates social science and medicine more longitudinally.

## Supplementary Information

**Additional file 1.** 4-week final report (2020–21).

**Additional file 2. **Rubric for evaluating the 4-week report on SDHs (2020–21).

## Data Availability

All data analyzed during the current study are available from the corresponding author on reasonable request.

## Abbreviations

SDH: Social determinants of health
CBME: Community-based medical education

## References

[1] World Health Organization. Social determinants of health. Available from: https://www.who.int/health-topics/social-determinants-of-health. Accessed 17 Nov 2022.

[2] Braveman P, Gottlieb L (2014). The social determinants of health: it's time to consider the causes of the causes. Public Health Rep.

[3] 2030 Healthy People. Social Determinants of Health. Available from: https://health.gov/healthypeople/priority-areas/social-determinants-health. Accessed 17 Nov 2022.

[4] Committee on Educating Health Professionals to Adress the Social Determinants of Health; Board on Global Health; Institute of Medicine; National Academies of Science, Engineering, and Medicine. A Framework for Educating Health Professionals to Address the Social Determinants of Health. Washington D.C.: National Academies Press; 2016. 27854400

[5] Siegel J, Coleman DL, James T (2018). Integrating social determinants of health into graduate medical education: A call for action. Acad Med.

[6] Royal College of Physicians and Surgeons of Canada. CanMEDS Framework. Available from: http://www.royalcollege.ca/rcsite/canmeds/canmeds-framework-e. Accessed 17 Nov 2022.

[7] Lewis JH, Lage OG, Grant BK, Rajasekaran SK, Gemeda M, Like RC, Santen S, Dekhtyar M (2020). Addressing the social determinants of health in undergraduate medical education curricula: a survey report. Adv Med Educ Pract.

[8] Martinez IL, Artze-Vega I, Wells AL, Mora JC, Gillis M (2015). Twelve tips for teaching social determinants of health in medicine. Med Teach.

[9] Campbell M, Liveris M, Caruso Brown AE, Williams A, Ngongo W, Persell S, Mangold KA, Adler MD (2022). Assessment and evaluation in social determinants of health education: a national survey of US medical schools and physician assistant programs. J Gen Intern Med.

[10] Doobay-Persaud A, Adler MD, Bartell TR. Teaching the social determinants of health in undergraduate medical education: a scoping review. J Gen Intern Med. 2019;34(5):720–30. 10.1007/s11606-019-04876-0 PMC6502919 30993619

[11] Ministry of education, culture, sports, science and technology. Medical education model core curriculum, revised 2017. (Japanese). Available from: https://www.mext.go.jp/component/b_menu/shingi/toushin/__icsFiles/afieldfile/2017/06/28/1383961_01.pdf. Accessed 3 Dec 2022.

[12] Ministry of education, culture, sports, science and technology. Medical education model core curriculum, revised 2022. Available from: https://www.mext.go.jp/content/20221202-mtx_igaku-000026049_00001.pdf. Accessed 3 Dec 2022.

[13] Ozone S, Haruta J, Takayashiki A, Maeno T, Maeno T (2020). Students’ understanding of social determinants of health in a community-based curriculum: a general inductive approach for qualitative data analysis. BMC Med Educ.

[14] Haruta J, Takayashiki A, Ozone S, Maeno T, Maeno T. How do medical students learn about SDH in the community? A qualitative study with a realist approach. Med Teach. 2022:44(10):1165–72. 10.1080/0142159X.2022.2072282 35583394

[15] Thomas DR (2006). A general inductive approach for analyzing qualitative evaluation data. Am J Eval.

[16] Aronson L (2011). Twelve tips for teaching reflection at all levels of medical education. Med Teach.

[17] University of Reading. Descriptive, analytical and reflective writing. Available from: https://libguides.reading.ac.uk/writing. Updated January 2, 2020. Accessed 17 Nov 2022.

[18] Hanton N, Smith D (1995). Reflection in teacher education: towards definition and implementation. Teach Teach Educ.

[19] World Health Organization. Social determinants of health, The solid facts. Second edition. Available from : http://www.euro.who.int/__data/assets/pdf_file/0005/98438/e81384.pdf. Accessed 17 Nov 2022.

[20] Michaeli D, Keough G, Perez-Dominguez F, Polanco-Ilabaca F, Pinto-Toledo F, Michaeli J, Albers S, Achiardi J, Santana V, Urnelli C, Sawaguchi Y, Rodríguez P, Maldonado M, Raffeeq Z, de Araujo MO, Michaeli T (2022). Medical education and mental health during COVID-19: a survey across 9 countries. Int J Med Educ.

[21] Arima M, Takamiya Y, Furuta A, Siriratsivawong K, Tsuchiya S, Izumi M (2020). Factors associated with the mental health status of medical students during the COVID-19 pandemic: a cross-sectional study in Japan. BMJ Open.

[22] Perez-Dominguez F, Polanco-Ilabaca F, Pinto-Toledo F, Michaeli D, Achiardi J, Santana V, Urnelli C, Sawaguchi Y, Rodríguez P, Maldonado M, Raffeeq Z, de Araujo MO, Rebolledo C (2021). Lifestyle changes among medical students during covid-19 pandemic: a multicenter study across nine Countries. Health Educ Behav.

[23] Tashiro T, Maeda N, Tsutsumi S, Komiya M, Arima S, Mizuta R, Fukui K, Nishikawa Y, Urabe Y (2022). Association between sedentary behavior and depression among Japanese medical students during the COVID-19 pandemic: a cross-sectional online survey. BMC Psychiatry.

