# Supplementary material for: Three-year evaluation of a program teaching social determinants of health in community-based medical education: a general inductive approach for qualitative data analysis
Source: BMC Med Educ. 2023 May 12;23:332. doi: 10.1186/s12909-023-04320-2 (PMC10176298; doi:10.1186/s12909-023-04320-2)
Supplement: Supplementary file 1 — Additional file 1. 4-week final report (2020–21). [file 12909_2023_4320_MOESM1_ESM.docx]

Additional file 1. 4-week final report (2020–21)

Answer each question based on your experience in the clerkship within the character limit.

Question 1.

Answer the following questions about what you learned from patients with whom you or healthcare professionals (not limited to physicians) interacted while referring to the evaluation criteria.

1. What was the patient’s background? What kinds of interactions did they have? How did the patient or family react? (Approximately 150–250 words.)
2. In relation to the case above, answer the following three questions in your own words. (Approximately 400–600 words in total.)
3. What did you learn about the significance of healthcare professionals asking patients about information other than their illness?
4. What did you learn about the significance of healthcare professionals understanding the characteristics of the family and community?
5. What role do you think healthcare professionals should play in the community with regard to individuals (patients, families, or the local community)?

Question 2.

Answer the following questions about the upstream social determinants of health you learned about in the clerkship, while referring to the evaluation criteria.

1. Describe your experience of learning about social determinants of health. (Approximately 200–300 words.)
2. In relation to the experience above, answer the following two questions in your own words. (Approximately 400–600 words in total)
3. What did you learn about the significance of healthcare professionals being aware of the social determinants of health?
4. To support the health of the community, what roles do you think healthcare professionals should play in the community?
