# Supplementary material for: Three-year evaluation of a program teaching social determinants of health in community-based medical education: a general inductive approach for qualitative data analysis
Source: BMC Med Educ. 2023 May 12;23:332. doi: 10.1186/s12909-023-04320-2 (PMC10176298; doi:10.1186/s12909-023-04320-2)
Supplement: Supplementary file 2 — Additional file 2. Rubric for evaluating the 4-week report on SDHs (2020–21). [file 12909_2023_4320_MOESM2_ESM.docx]

Additional file 2. Rubric for evaluating the 4-week report on SDHs (2020–21)

| Theme | Evaluation criteria | Excellent (A+) | Good (A) | Fair (B) | Needs  Improvement (C) | Fail (D) |
| --- | --- | --- | --- | --- | --- | --- |
| Understanding  SDHs | 1. Describes the cases related to SDHs concretely. 2. Based on the above experience, describes the significance of being aware of SDHs. 3. Describes in one’s own words the role that healthcare professionals (including non-physicians) should play in supporting the health of the community. | From the experience in the community, becomes aware of SDHs and fully considers how each factor affects health over time.  In addition, clearly verbalizes the significance of healthcare professionals in the community understanding SDHs and of approaching both individuals and the community. | From the experience in the community, becomes aware of SDHs and describes how each factor affects health, including the time course for various cases.  In addition, describes the significance and roles in the community of healthcare professionals who understand SDHs. | From the experience in the community, becomes aware of SDHs and describes them. In addition, describes the significance of healthcare professionals to addressing these factors. | Insufficient description of the experience in the community, or poor understanding of SDHs. | The requested content is not described. |

SDH: social determinant of health.
